# Inhibition of *Pseudomonas aeruginosa* secreted virulence factors reduces lung inflammation in CF mice

**DOI:** 10.1080/21505594.2018.1489198

**Published:** 2018-07-27

**Authors:** Angela Sandri, Alessia Ortombina, Federico Boschi, Eleonora Cremonini, Marzia Boaretti, Claudio Sorio, Paola Melotti, Gabriella Bergamini, Maria Lleo

**Affiliations:** a Department of Diagnostics and Public Health, University of Verona, Verona, Italy; b Department of Computer Science, University of Verona, Verona, Italy; c Department of Medicine, University of Verona, Verona, Italy; d Cystic Fibrosis Center, Azienda Ospedaliera Universitaria Integrata di Verona,Verona, Italy

**Keywords:** Cystic fibrosis, *Pseudomonas aeruginosa*, virulence factors, clarithromycin, protease inhibitors, lung inflammation, in vivo imaging

## Abstract

**Background**: Cystic fibrosis (CF) lung infection is a complex condition where opportunistic pathogens and defective immune system cooperate in developing a constant cycle of infection and inflammation. The major pathogen, *Pseudomonas aeruginosa*, secretes a multitude of virulence factors involved in host immune response and lung tissue damage. In this study, we examined the possible anti-inflammatory effects of molecules inhibiting *P. aeruginosa* virulence factors.

**Methods**: Pyocyanin, pyoverdine and proteases were measured in bacterial culture supernatant from different *P. aeruginosa* strains. Inhibition of virulence factors by sub-inhibitory concentrations of clarithromycin and by protease inhibitors was evaluated. Lung inflammatory response was monitored by in vivo bioluminescence imaging in wild-type and CFTR-knockout mice expressing a luciferase gene under the control of a bovine IL-8 promoter.

**Results**: The amount of proteases, pyocyanin and pyoverdine secreted by P. aeruginosa strains was reduced after growth in the presence of a sub-inhibitory dose of clarithromycin. Intratracheal challenge with culture supernatant containing bacteria-released products induced a strong IL-8-mediated response in mouse lungs while lack of virulence factors corresponded to a reduction in bioluminescence emission. Particularly, sole inactivation of proteases by inhibitors Ilomastat and Marimastat also resulted in decreased lung inflammation.

**Conclusions**: Our data support the assumption that virulence factors are involved in *P. aeruginosa* pro-inflammatory action in CF lungs; particularly, proteases seem to play an important role. Inhibition of virulence factors production and activity resulted in decreased lung inflammation; thus, clarithromycin and protease inhibitors potentially represent additional therapeutic therapies for *P. aeruginosa*-infected patients.

## Introduction

Cystic fibrosis (CF) lung infection is a complex condition where opportunistic pathogens and defective immune system cooperate in developing a constant cycle of infection and inflammation [1]. *Pseudomonas aeruginosa* causes chronic respiratory infections in more than 50% of adult CF patients, therefore it is considered the main respiratory pathogen [2]. A period of initial intermittent, recurrent lung colonization is described, when antibiotic treatment can temporarily eradicate the infection. This phase can last for years but often transition into a chronic infection occurs, inducing a state of chronic inflammation [3]. Indeed, increased number of neutrophils, alveolar macrophages and T lymphocytes were found in alveoli of explanted lungs from infected CF patients [4]. Despite the inflammatory response and intensive antibiotic therapy, most infections caused by *P. aeruginosa* persist for long time, eventually leading to respiratory failure and lung transplantation or death [2].

Especially during early infection, *P. aeruginosa* expresses a wealth of virulence factors exhibiting strong pro-inflammatory properties [5]. Among these, proteases can disrupt lung tissue and modulate host inflammatory response [6–8]; the blue-green pigment pyocyanin causes host cells oxidative stress and dysregulates immune mechanisms [9–11]; the siderophore pyoverdine is both able to sequester iron from host depots and to regulate bacterial virulence [12,13]. In a previous study, we observed that macrolide antibiotic azithromycin (AZM) acts on *P. aeruginosa* by reducing the synthesis of proteases and other exoproducts involved in bacterial virulence and the associated host inflammatory response. Indeed, AZM is known to interact with the 50S ribosomal subunit and affect specific genes and transcriptional factors involved in the regulation of virulence [14]. This inhibitory action was associated with a decrease of lung immune response in mice with beneficial effects for the animals in terms of reduced inflammation [15], suggesting that bacterial virulence down-regulation might be a promising anti-inflammatory strategy.

Patients with chronic *P. aeruginosa* lung infection are often treated with AZM because of its anti-pseudomonal and immunomodulatory properties [16,17]. Unfortunately, there is a number of patients that do not benefit from AZM therapy or that show adverse effects to the drug [18]. Especially for these patients, it is important to find alternative treatments. In the last 15 years, various studies were conducted to evaluate therapy with clarithromycin (CLM), another macrolide antibiotic. Although the comparison of the outcomes of these studies is limited by the different treatment regimens, doses, drug formulations and clinical factors evaluated, low-dose CLM seems to be more effective, as supported also by its low-dose benefits in the treatment of diffuse panbronchiolitis which shares many similarities in clinical and pathological characteristics with CF [19–23]. Moreover, CLM treatment was shown to decrease lung inflammatory processes and chronic airways hypersecretion in non-CF patients with bronchiectasis [24,25]. Pertaining to its anti-pseudomonal effects, CLM has no bactericidal activity against *P. aeruginosa*, like other macrolides, but can interfere with protein synthesis and inhibit protease expression, twitching motility and biofilm maturation, promoting biofilm permeability and favoring penetration of other antimicrobial agents like ciprofloxacin and tobramycin, also used in CF therapy [26–28].

Although the combination of anti-bacterial and anti-inflammatory action in a single molecule might seem particularly appealing, the use of antibiotics also favors the selection of resistant bacteria, limiting the long-term use of these molecules. Indeed, AZM shows beneficial effects in the first year of therapy while the reduced efﬁcacy associated with longer treatment might be due to development of bacterial resistance [29]. Therefore, in addition to the development of new antibiotics, also alternative, non-antibiotic anti-inflammatory therapies, targeting the damaging and pro-inflammatory effects of virulence factors like proteases, might help to face this concern [30]. The most potent anti-protease molecules are hydroxamate-based broad spectrum matrix metalloprotease inhibitors (MMPIs), which mimic collagen structure, thus binding to the enzyme active site and inactivating it by chelation of the catalytic zinc ion. Interestingly, their inhibitory effect can apply also to bacterial metalloproteases, as it has been demonstrated by Ilomastat (Galardin, GM6001) ability to inhibit thermolysin and *P. aeruginosa* elastase [31]. Ilomastat reached phase III clinical trials as therapy for corneal ulcers and underwent pre-clinical development as topical post-injury treatment for chemical burns, as therapy for diabetic retinopathy and cancer and as inhaled treatment for chronic obstructive pulmonary disease [32–34]. The first MMPI to be clinically tested was Batimastat, an injectable drug, rapidly abandoned in favor of the newer, orally available analogue Marimastat which also entered clinical trials as anticancer agent (glioblastoma, breast, ovarian, pancreatic, gastric, small and non-small cell lung cancers). Marimastat showed a favorable pharmacokinetic profile, high systemic bioavailability, linear dose-plasma relationship, balanced excretion (75% hepatic, 25% renal), an elimination half-life compatible with twice-daily dosing and modest efficacy in delaying disease progression. However, significance could not be established due to dose-limiting toxicity, identified with appearance of musculoskeletal symptoms reversible upon drug discontinuation [35,36]. The intense clinical development of these molecules, associated with the opportunity to target proteases involved in chronic infection processes, suggest their possible application to respiratory infectious diseases like CF lung infection.

In this study, we investigated anti-virulence properties of CLM and MMPIs against *P. aeruginosa* and their beneficial effects on lung inflammation in mice.

## Results

### Clarithromycin reduces secreted virulence factors

**Figure 1.**
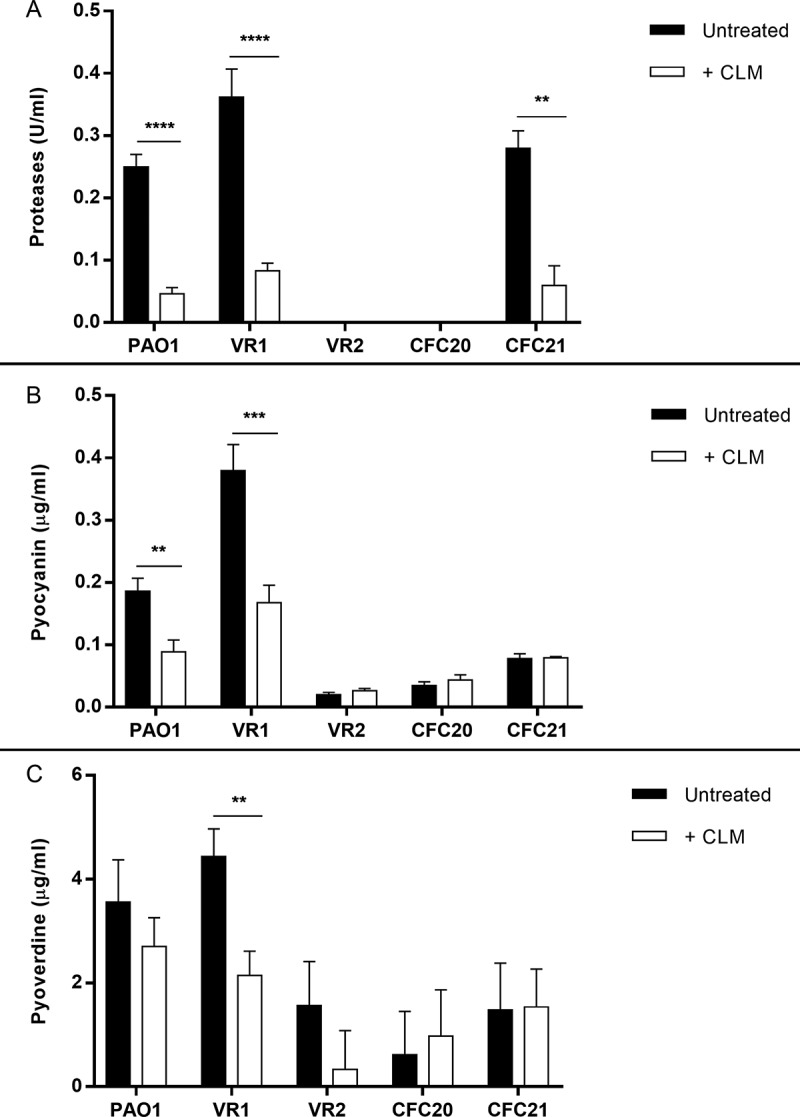
Proteases (A), pyocyanin (B) and pyoverdine (C) measured in culture supernatant collected from *P. aeruginosa* strains grown in absence/presence of 45 ug/ml CLM. Each value represents the mean ± SEM of 3 experiments. Statistical analysis was performed by Mann-Whitney test; *p < 0.05, **p < 0.01, ***p < 0.001 and **** p < 0.0001.

*P. aeruginosa* strains secrete various amounts of proteases, pyocyanin and pyoverdine, as measured in their culture supernatant. Particularly, VR1 strain, isolated from a patient with intermittent infection, showed ability to secrete higher amounts of virulence factors in comparison to laboratory strain PAO1 and other clinical strains from both intermittently and chronically infected patients (Suppl. Figure 1). Performing MIC assay, we established that doses up to 256 µg/ml CLM had no bacteriostatic activity against the selected strains (Table 1). Thus, we measured proteases, pyocyanin and pyoverdine in culture supernatant of bacteria grown in presence of a sub-MIC dose of CLM (45 µg/ml). As shown in Figure 1, this treatment caused the reduction of the levels of proteases in all protease-secreting strains, while the inhibitory effect on pyoverdine and pyocyanin varied among the tested strains. Particularly, VR1 strain showed a significant reduction of all the three virulence factors, similar to what we previously observed with AZM [15]. Iron levels in culture medium seem not to interfere with CLM effect on pyoverdine (Suppl. Figure 2).

**Table 1.** Description of *P. aeruginosa* strains and CLM antimicrobial activity.

Bacterial strain	Origin	Phenotype	MIC (µg/ml)
PAO1	Laboratory strain	Non Mucoid	> 256
VR1	CF intermittent infection	Non Mucoid	> 256
VR2	CF intermittent infection	Mucoid	> 256
CFC20	CF chronic infection	Non Mucoid	> 256
CFC21	CF chronic infection	Non Mucoid	> 256

**Figure 2.**
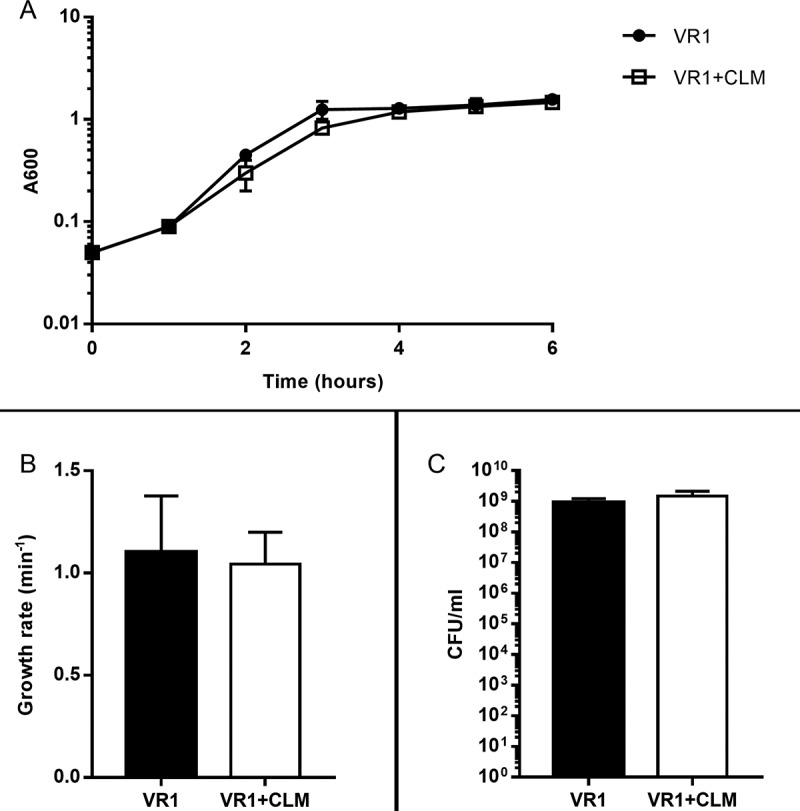
Growth curves (A), growth rate (B) and CFU count (C) measured from VR1 growth in absence/presence of 45 ug/ml CLM. Absorbance at 600 nm was measured every hour for 6 hours (A) and growth rate per minute was calculated from exponential phase of growth curves (B). CFU were counted on TSA after plating serial dilutions of inocula (C). Each value represents the mean ± SEM of 3 experiments.

This inhibitory effect does not concern bacterial growth; indeed, CLM did not affect growth rate and cell viability of VR1, the strain most inhibited by the macrolide in terms of virulence factors secretion (Figure 2). Thus, probably by interfering with the production of *P. aeruginosa* exoproducts as previously shown by AZM inhibiting metalloproteases synthesis [15], CLM could reduce bacterial virulence and indirectly modulate virulence-induced host inflammatory response.

#### Culture supernatant lacking virulence factors does not induce inflammation in mouse lungs

**Figure 3.**
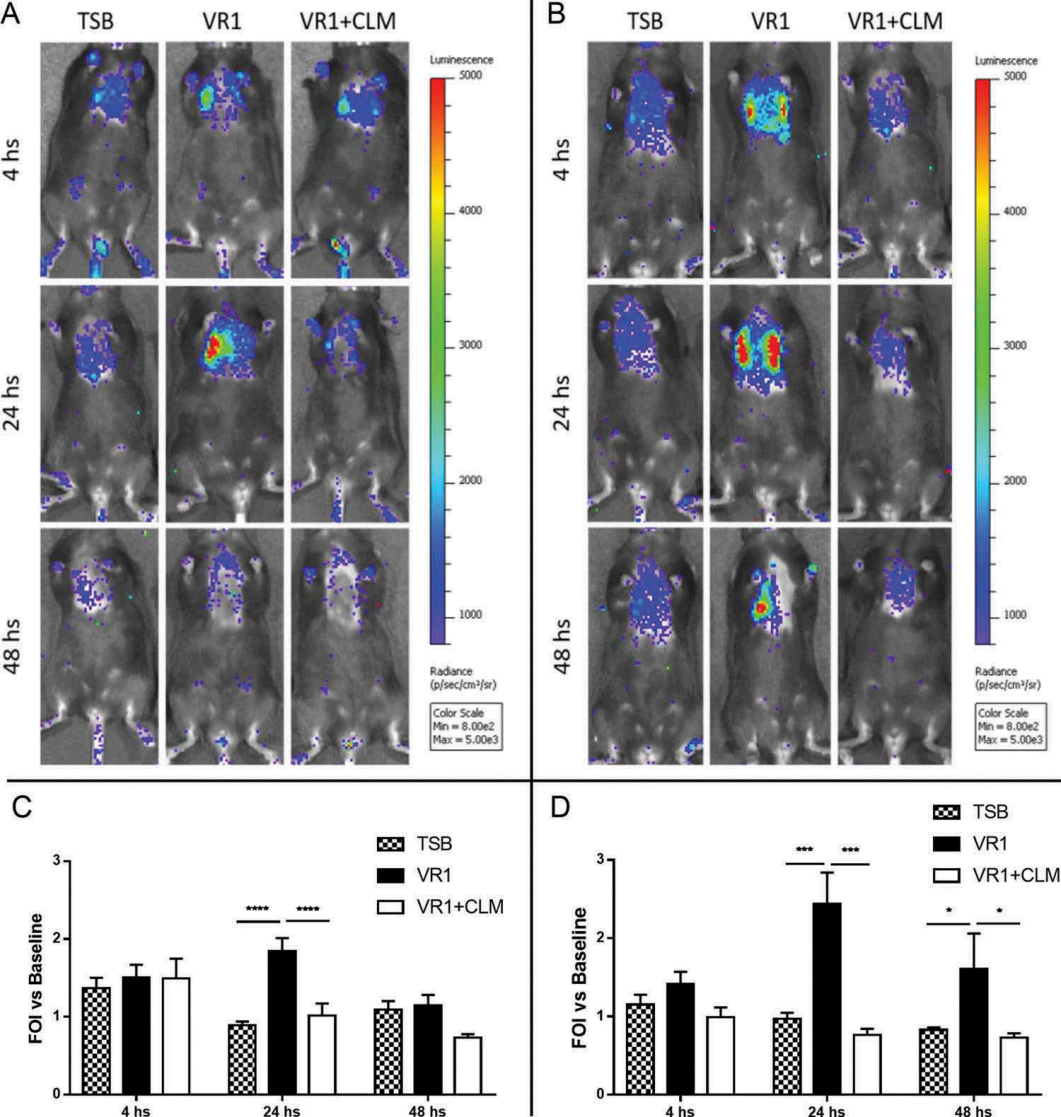
Representative images (upper panels) and photon emission measurement (lower panels) of bioluminescence emission in WT (A, C) and CF (B, D) mice challenged with concentrated culture supernatant from VR1 grown in absence/presence of 45 ug/ml CLM. Control mice were treated with TSB. Photon emission is expressed as folds of increase (FOI) vs. baseline. Each value represents the mean ± SEM of 6 animals (biological replicates) per group. Statistical analysis was performed by 2way ANOVA followed by Dunnett’s multiple comparisons test; *p < 0.05, ***p < 0.001, **** p < 0.0001.

To evaluate *in vivo* the possible reduction of inflammation associated with inhibition of *P. aeruginosa* virulence exoproducts, we used a transgenic mouse model that was previously validated [15,37,38]. In presence of lung inflammatory response induced by non-invasive intratracheal challenge with culture supernatant, the exogenous IL-8 promoter is recognized by the mouse transcriptional apparatus and luciferase enzyme is produced. At 4, 24 and 48 hours after treatment, bioluminescence emission in the chest region was recorded following D-luciferin injection. As shown in Figure 3, in both WT and CF animals the challenge with VR1 culture supernatant induced after 24 hours a strong inflammatory response in terms of IL-8 reporter activation. The bioluminescence signal lasted longer in CF mice, whose emission after 48 hours was still significantly higher than in mice challenged with the control medium. Interestingly, in both animal strains, the supernatant collected from VR1 grown in presence of 45 µg/ml CLM (VR1+ CLM) and containing lower levels of virulence factors provoked only a minor response: the average of photons emitted was significantly lower than in mice challenged with VR1 supernatant and comparable to the bioluminescence signal induced by the control medium.

#### Protease inhibition reduces inflammation in mouse lungs

**Figure 4.**
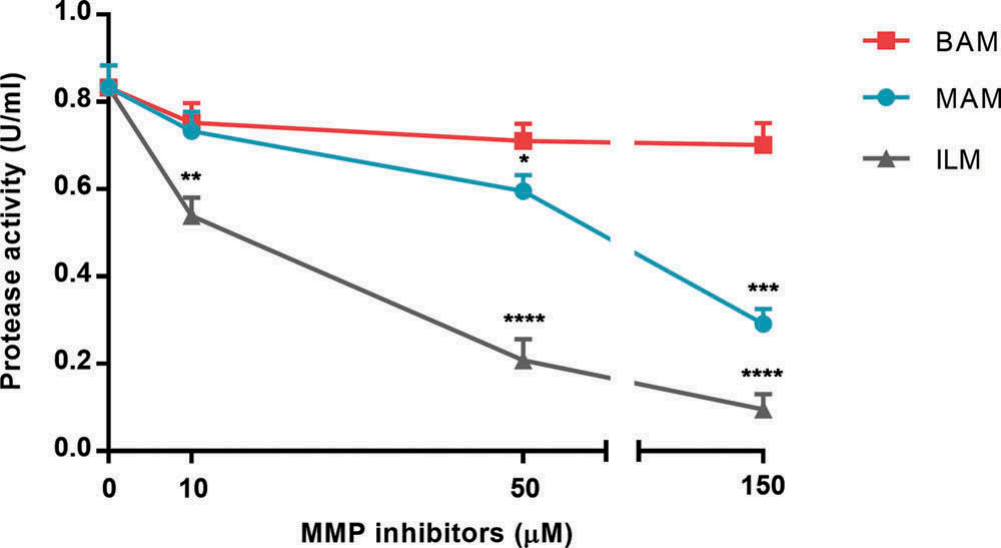
Protease activity in VR1 concentrated culture supernatant was measured in presence of MMPIs Marimastat (MAM), Batimastat (BAM) and Ilomastat (ILM). Each value represents the mean ± SD of 3 experiments. Statistical analysis was performed by Kruskal-Wallis test and Dunn’s multiple comparisons test; * p < 0.05, **p < 0.01, ***p < 0.001, **** p < 0.0001 vs. non-treated supernatant (0 µM MMPIs).

Focusing on a specific family of virulence factors, we evaluated the ability of human MMPIs Ilomastat, Batimastat and Marimastat to inhibit *P. aeruginosa* proteases secreted in VR1 culture supernatant. As shown in Figure 4, Ilomastat had the best inhibitory effect, producing a strong inactivation of bacterial proteases already at 50 µM and reaching 89% inhibition at 150 µM. At 150 µM, also Marimastat induced a 65% inhibition of the protease activity, whereas Batimastat had poor inhibitory effect on bacterial proteases even at higher concentrations (up to 300 µM, data not shown).

**Figure 5.**
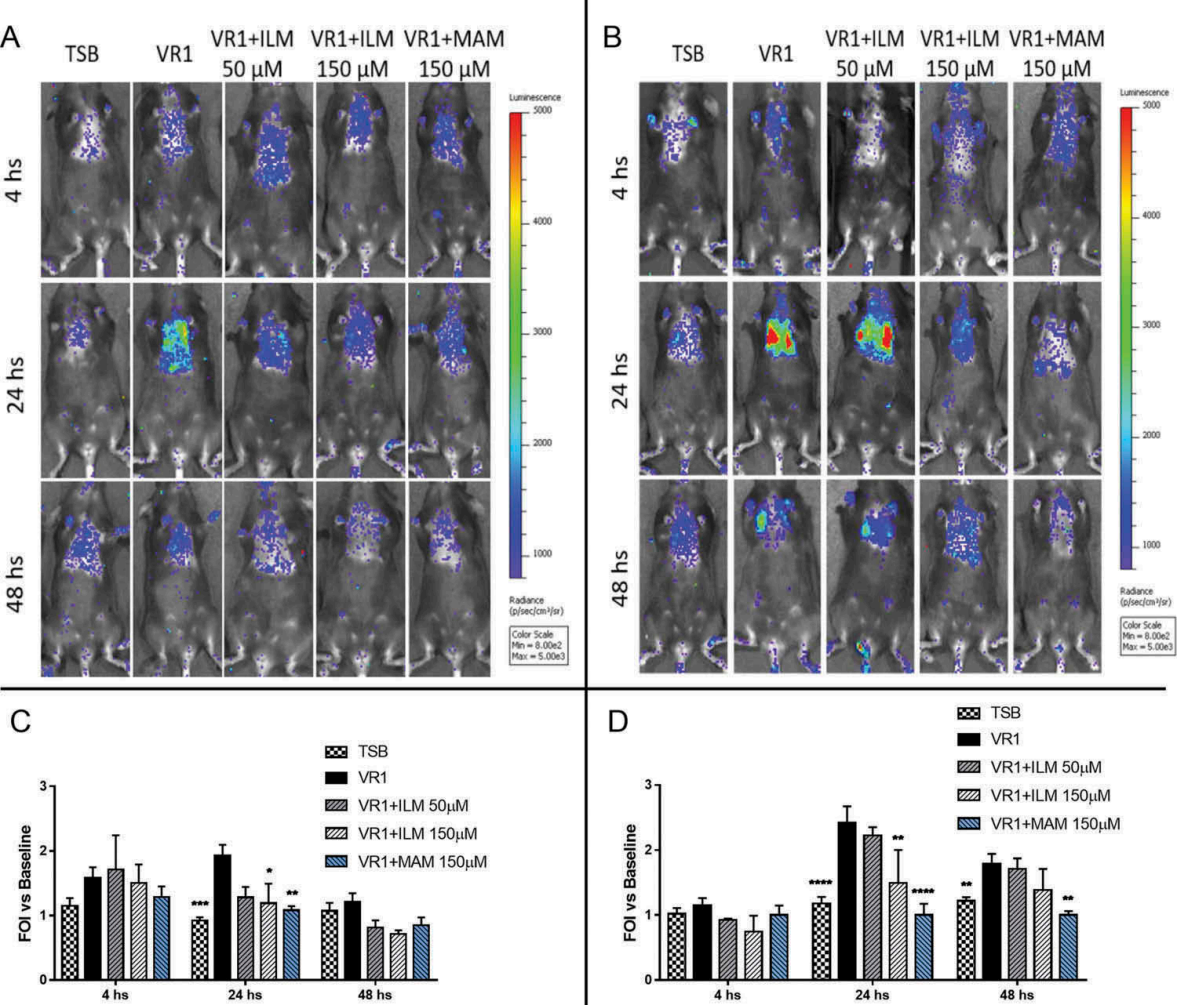
Representative images (upper panels) and photon emission measurement (lower panels) of bioluminescence emission in WT (A, C) and CF (B, D) mice challenged with VR1 concentrated culture supernatant pre-treated with 50 and 150 µM Ilomastat (ILM) and 150 µM Marimastat (MAM). VR1 supernatant was used as positive control (black), TSB was used as negative control. Photons emission is expressed as folds of increase (FOI) vs. baseline. Each value represents the mean ± SEM of 6 animals (biological replicates) per group. Statistical analysis was performed by 2way ANOVA followed by Dunnett’s multiple comparisons test; *p < 0.05, **p < 0.01, ***p < 0.001, **** p < 0.0001.

To evaluate *in vivo* the possible reduction of inflammation associated with inhibition of proteases, we monitored the IL-8 reporter activation induced by non-invasive intratracheal challenge with VR1 culture supernatant pre-treated with Ilomastat and Marimastat by in vivo imaging. As shown in Figure 5, protease inhibition with 50 µM Ilomastat could slightly reduce the bioluminescence signal only in WT mice but was insufficient in CF mice. Increasing the dose to 150 µM, a significantly diminished emission was observed in both mouse strains at 24 hours after the challenge. Regarding Marimastat, the same concentration (150 µM) was even more effective, since bioluminescence emission in CF mice was still significantly reduced at 48 hours after the intratracheal challenge with the pre-treated supernatant.

## Discussion

Patients with chronic *P. aeruginosa* lung infection are often treated with low-dose AZM, a macrolide antibiotic known to interact with the 50S ribosomal subunit and affect specific genes and transcriptional factors involved in the regulation of virulence [14]. AZM has no bactericidal activity against *P. aeruginosa* but previously showed the ability to reduce secreted virulence factors such as pyocyanin, pyoverdine and proteases, due to its inhibitory effect on protein synthesis [15]. The observed anti-virulence activity is likely to be involved in the clinical benefits associated with AZM treatment in the first year of therapy while the reduced efﬁcacy associated with longer treatment duration might be due to development of bacterial resistance to AZM anti-virulence activity. Thereby, evaluation of molecules with similar antibacterial mechanisms is important to find alternative treatments for patients not responding to AZM or for whom the treatment lost efficacy during time [18,29].

Macrolide CLM already showed clinical effectiveness in treating diffuse panbronchiolitis and has been studied as therapy for other respiratory diseases [23–25]; CLM effects in CF patients were also evaluated in different studies, although with contrasting outcomes [19–22]. Like AZM, low-dose CLM has no bactericidal activity against *P. aeruginosa*, but we observed that it can lower the amount of virulence factors secreted by *P. aeruginosa* and reduce the associated host inflammatory response in both WT and CF mice. Although our results seem in contrast with previous findings [39] reporting higher mortality rate in mice infected with macrolide-treated *P. aeruginosa*, the different challenge must be considered. Our investigation is focused on secreted exoproducts, while the infection process involves other factors, like biofilm formation. Indeed, CLM is known to inhibit maturation of established biofilm but not its formation [26,40], which might partially explain the observations from Kobayashi and colleagues.

Our results support that CLM acts similarly to AZM, thus it might represent a candidate therapy for treating CF patients with chronic infection. We encourage more clinical trials to assess the effectiveness of this therapy, which gain particular interest when considering the possibility of local aerosol administration; indeed, its formulation as dry powder inhaler is currently under investigation [28]. To our knowledge, CLM treatment has never been tested in patients that already showed no improvement under AZM therapy. A clinical study in this group of patients might clarify the possible use of CLM as an alternative therapy and help to understand the involvement of *P. aeruginosa* resistance in AZM long-term treatment ineffectiveness.

Among various virulence factors, proteases from both host and bacteria play a critical role in CF lung disease, contributing to lung tissue damage and exacerbation of the inflammatory response. *P. aeruginosa* exogenous proteases such as AprA and LasB can alter mucociliary clearance, degrade lung tissue and dysregulate host immune system, thus strongly contributing to lung disease [6]. We previously showed that *P. aeruginosa* proteases are major exoproducts of selected clinical strains and that they might interfere with the host immune system by down-regulating CXCR1 on the surface of human neutrophils [41]. Downregulation of protease production is part of the anti-virulence effects of macrolides and is likely to be involved in their anti-inflammatory effect associated with virulence inhibition. Thus, molecules targeting proteases might represent an alternative or complementary anti-inflammatory therapy in CF.

Among many classes of protease-inhibiting compounds, broad spectrum hydroxamates are attractive candidates, due to their intense clinical evaluation during the 90’s when many members entered clinical trials as cancer therapeutics [35,36]. Hydroxamate-based broad spectrum MMPI Ilomastat had previously been reported to inhibit *P. aeruginosa* elastase [31]; indeed, we confirmed that Ilomastat and its analogue Marimastat can reduce the protease activity in *P. aeruginosa* culture supernatant and this mechanism seems associated with anti-inflammatory effects in mice. Although in vitro Ilomastat showed a greater inhibitory effect on proteases in comparison to Marimastat, in vivo Marimastat was more effective in reducing lung inflammation. These discrepancies might be due to MMPIs effects on the host side. Indeed, MMPIs can inhibit also host proteases and have been reported to interact with host inflammatory response mechanisms like neutrophil and macrophage recruitment [34,42]. Although we cannot address a specific target, the possibility of an additional anti-inflammatory activity elicited by Marimastat might represent a further advantage of anti-protease therapy.

We cannot exclude that inhibition of host proteases might also participate in the observed beneficial effects; nevertheless, inhibition of host proteases like neutrophil elastase, thought to be the main source of protease activity in CF lungs, might represent an additional benefit of these molecules. These results highlight the key role played by proteases in host inflammatory response and suggest protease inhibition as an alternative strategy to dampen the inflammatory response and concomitant airways damage. Particularly, inhaled therapy with MMPIs [34] might avoid the side-effects reported with oral Marimastat and potentially represent a new local treatment for CF patients.

In conclusion, the results from our study highlight the important role played by *P. aeruginosa* virulence factors in CF host inflammatory response, support the inhibition of these factors as a potential therapeutic strategy and encourages the development of treatments targeted against bacterial virulence.

## Material and methods

### Bacterial strains

*P. aeruginosa* laboratory strain PAO1 and clinical strains VR1, VR2, CFC20, CFC21 [15,43] were used in this study. Clinical isolates were previously collected from sputum samples of patients followed at the Cystic Fibrosis Center of Verona, Italy. VR1 and VR2 strains were isolated from intermittently infected patients; CFC20 and CFC21 strains were isolated from chronically infected patients. VR2 is characterized by mucoid morphology. Strains were stored in Microbank^TM^ (Biolife Italiana, cat. n. 17PL170M) at −80°C.

#### Minimum inhibitory concentration (MIC) assay

*P. aeruginosa* strains were streaked on tryptic soy agar (TSA, Thermo Fisher Scientific, Oxoid^TM^, cat. n. CM0131R) plates and incubated at 37°C for 24–48 hours. 1–2 colonies were inoculated in 10 ml tryptic soy broth (TSB, Thermo Fisher Scientific, Oxoid^TM^, cat. n. CM0129R) shaking at 37°C for 16 hours. Optical density (OD) at 600 nm was measured using a spectrophotometer, cultures were diluted to 0.05 OD/ml and inoculated in 96-wells microtiter plates with increasing concentrations of CLM (Klaricid, Abbott). After incubation at 37°C for 16 hours, the MIC values were identified as the lowest drug concentration causing a complete inhibition of bacterial growth.

#### Growth curves

VR1 strain was plated on TSA plates and incubated at 37°C for 24–48 hours. 1–2 colonies were inoculated in 10 ml TSB shaking at 37°C overnight. OD_600_ was measured, culture was diluted to 0.05 OD/ml in 50 ml TSB with/without sub-MIC CLM (45 µg/ml) and incubated at 37°C shaking. OD_600_ was measured every hour and growth rate was calculated using GraphPad Prism 7.0 software. After 6 hours, cultures were serially diluted and plated on TSA. Plates were incubated at 37°C for 24 hours and colony forming units (CFU) were counted.

#### Culture supernatant collection and concentration

*P. aeruginosa* strains were streaked on TSA plates and incubated at 37°C for 24–48 hours. 1–2 colonies were inoculated in 30 ml TSB shaking at 37°C for 16 hours. OD_600_ was measured and cultures were diluted to 0.1 OD/ml in 30 ml of the same medium, with/without sub-MIC CLM (45 µg/ml). After shaking at 37°C for 16 hours, cultures were diluted to 0.2 OD/ml and centrifuged at 7000 g for 30 min at 4°C. Supernatants were collected and sterile-filtered. For mice intratracheal challenge, supernatants were 10X-concentrated using 30 kDa cut-off Amicon Ultra-15 centrifugal filter units (Merck Millipore, cat. n. UFC903024) pre-coated with 10 mg/ml bovine serum albumin (Sigma-Aldrich, cat. n. A2153). Concentrates were sterile-filtered and stored at −20°C.

#### Azocasein assay

Protease activity in culture supernatants was determined by azocasein assay. Briefly, 350 µl reaction mixture containing 0.1 M Tris-HCl pH 8.0 and 1% azocasein (Sigma-Aldrich, cat. n. A2765) resuspended in 0.5% NaHCO_3_ was added to 150 µl supernatant with/without MMPIs Ilomastat, Marimastat, Batimastat (Sigma-Aldrich, cat. n. M5939, M2699, SML0041) and incubated at 37°C for 20 minutes shaking. After addition of 1 ml 7% ice-cold perchloric acid, the solution was centrifuged. 150 µl 10 N sodium hydroxide were added to the clear supernatant and OD at 430 nm was measured. One protease unit was calculated as the amount of enzyme producing an increase of 1 OD unit per hour.

#### Pyocyanin measurement

Pyocyanin was chloroform-extracted from culture supernatants. Briefly, 3 ml chloroform were added to 5 ml supernatant and the lower phase was mixed with 1 ml 0.2 M hydrogen chloride. OD at 530 nm was measured in the upper phase and pyocyanin concentration was calculated by multiplying for its ɛ^−1^ (17,072 µg/ml cm) and normalizing for the optical distance.

#### Pyoverdine measurement

Pyoverdine was measured by OD at 405 nm in culture supernatants and pyoverdine concentration was calculated using its ɛ (19 mM^−1^ cm^−1^) and normalizing for the optical distance.

#### Experimental animals

Female congenic C57BL/6J wild-type (WT) and gut-corrected CFTR^tm1UNC^ (CF) mice (8–10 weeks old) were purchased from Cystic Fibrosis animal Core facility (San Raffaele Hospital, Milan, Italy). Animals were maintained under conventional housing conditions. Prior to use, animals were acclimatized for at least 5–7 days to the local vivarium conditions having free access to standard rodent chow and tap water. Animal experiments were conducted in compliance with national (Decreto Legislativo n. 26, 4 Marzo 2014) and international laws and policies (Guide for the Care and Use of Laboratory Animals).

#### Reporter construct

Experimental animals were transfected with the bIL-8-Luc construct, containing a luciferase gene under the control of bovine IL-8 promoter (kindly provided by Prof. Gaetano Donofrio, University of Parma, Italy) [44]. Plasmid was transformed in competent *E. coli* DH5α cells by heat shock and purified by Qiagen Plasmid Maxi Kit (Qiagen, cat. n. 12,163) followed by phenol:chloroform extraction with precipitation in isopropanol and 70% ethanol. Plasmid concentration and purity were assessed using NanoDrop 2000c spectrophotometer (Thermo Fisher Scientific).

#### In vivo gene delivery

In vivo JetPEI (Polyplus Transfection, cat. n. 201-50G) was used as carrier for delivering bIL-8-Luc construct to lung tissue. As previously described [37], DNA and JetPEI were mixed with a final N/P ratio of 7–7.5 following manufacturer’s instructions. Briefly, 38–42 µg DNA and 5.3–6.3 µl JetPEI were separately diluted in 200 µl 5% glucose, mixed, and incubated at room temperature for 15 minutes. 400 µl of the mixture were intravenously injected through the tail vein after warming the animals for 5 minutes under a heating lamp. Expression and inactivation of the reporter were monitored by in vivo bioluminescence imaging after 24 hours and 7 days, respectively.

#### Intratracheal challenge

BIL-8-Luc transgenic mice were intratracheally challenged as previously described [37] with 10X-concentrated VR1 culture supernatant collected after growth with/without sub-MIC CLM (45 µg/ml) or MMPIs-treated/free. Briefly, mice were anesthetized with 2.5% isoflurane and placed on an intubation platform hanging by their incisor teeth. After visualization of the opening of the trachea using a laryngoscope, 50 µl stimulus were instilled by an intubation tube connected to a pressure control system. After 4, 24 and 48 hours, reporter activation was monitored by in vivo imaging.

#### In vivo bioluminescence imaging

Bioluminescence imaging of experimental animals was performed as previously described [37], using IVIS Spectrum imaging system (PerkinElmer). 10 minutes prior to bioluminescence recording, mice were anesthetized with 2.5% isoflurane and intraperitoneally injected with 150 mg/kg D-Luciferin (PerkinElmer, cat. n. 122,799). After 5 minutes-long luminescence recording, photons emitted from chest region were quantified using Living Image software (PerkinElmer).

#### Statistical analysis

Statistical analysis was performed using GraphPad Prism 7.0 software. Virulence factors measurements were analyzed by Mann-Whitney test, protease inhibition by MMPIs was analyzed by Kruskal-Wallis test followed by Dunn’s multiple comparisons test, and mice bioluminescence emission was analyzed by 2way ANOVA followed by Dunnett’s multiple comparisons test.

## Abbreviations

CF: cystic fibrosis
AZM: azithromycin
CLM: clarithromycin
MMPI: matrix metalloprotease inhibitor
TSA: tryptic soy agar
TSB: tryptic soy broth
