# Supplementary material for: Inhibition of Pseudomonas aeruginosa secreted virulence factors reduces lung inflammation in CF mice
Source: Virulence. 2018 Jul 27;9(1):1008–18. doi: 10.1080/21505594.2018.1489198 (PMC6086295; doi:10.1080/21505594.2018.1489198)
Supplement: Supplemental Material [file kvir-09-01-1489198-s001.docx]

**Supplementary files**


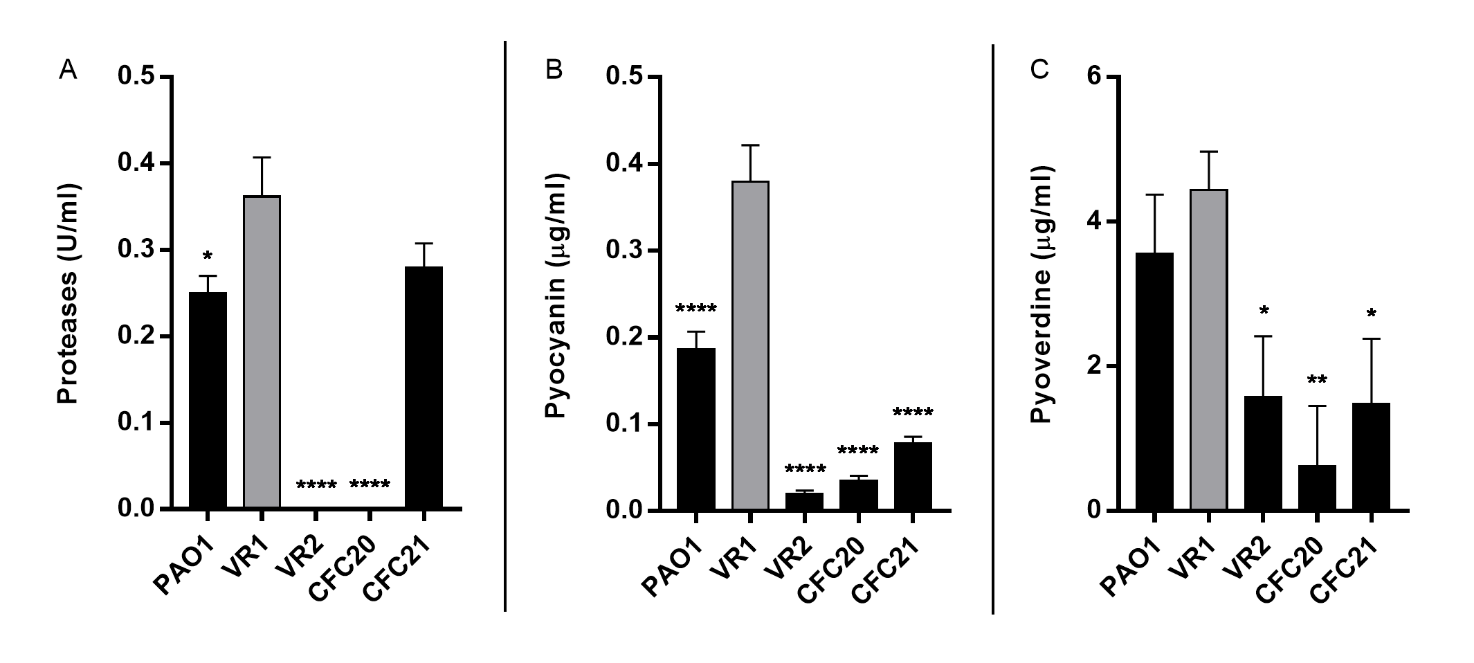


**Figure 1S:** Proteases (A), pyocyanin (B) and pyoverdine (C) measured in culture supernatant collected from *P. aeruginosa* strains. Each value represents the mean ± SEM of 3 experiments. Statistical analysis was performed by 1way ANOVA followed by Dunnett’s multiple comparisons test; *p<0.05, **p<0.01 and **** p<0.0001 vs. VR1 (grey bar).


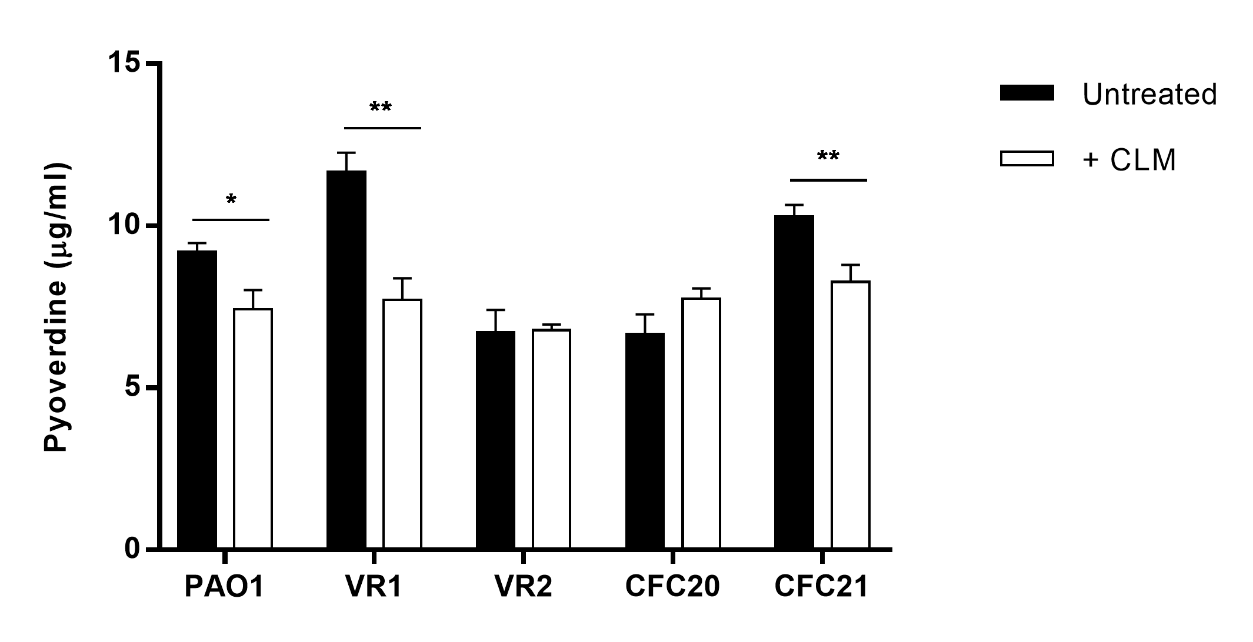


**Figure 2S:** Pyoverdine measured in culture supernatant collected from *P. aeruginosa* strains grown in King’s broth (low iron medium) in absence/presence of 45 ug/ml CLM. Each value represents the mean ± SEM of 3 experiments. Statistical analysis was performed by 1way ANOVA followed by t test; *p<0.05 and **p<0.01.
